# Local government responses for COVID-19 management in the Philippines

**DOI:** 10.1186/s12889-021-11746-0

**Published:** 2021-09-21

**Authors:** Dylan Antonio S. Talabis, Ariel L. Babierra, Christian Alvin H. Buhat, Destiny S. Lutero, Kemuel M. Quindala, Jomar F. Rabajante

**Affiliations:** 1 Institute of Mathematical Sciences and Physics, University of the Philippines Los Baños, Laguna, Philippines; 2 University of the Philippines Resilience Institute, University of the Philippines, Quezon City, Philippines; 3 Faculty of Education, University of the Philippines Open University, Laguna, Philippines

**Keywords:** COVID-19, Local government, Policies, Outlier, Quantitative methods

## Abstract

**Background:**

Responses of subnational government units are crucial in the containment of the spread of pathogens in a country. To mitigate the impact of the COVID-19 pandemic, the Philippine national government through its Inter-Agency Task Force on Emerging Infectious Diseases outlined different quarantine measures wherein each level has a corresponding degree of rigidity from keeping only the essential businesses open to allowing all establishments to operate at a certain capacity. Other measures also involve prohibiting individuals at a certain age bracket from going outside of their homes. The local government units (LGUs)–municipalities and provinces–can adopt any of these measures depending on the extent of the pandemic in their locality. The purpose is to keep the number of infections and mortality at bay while minimizing the economic impact of the pandemic. Some LGUs have demonstrated a remarkable response to the COVID-19 pandemic. The purpose of this study is to identify notable non-pharmaceutical interventions of these outlying LGUs in the country using quantitative methods.

**Methods:**

Data were taken from public databases such as Philippine Department of Health, Philippine Statistics Authority Census, and Google Community Mobility Reports. These are normalized using Z-transform. For each locality, infection and mortality data (dataset *Y*) were compared to the economic, health, and demographic data (dataset *X*) using Euclidean metric *d*=(*x*−*y*)^2^, where *x*∈*X* and *y*∈*Y*. If a data pair (*x*,*y*) exceeds, by two standard deviations, the mean of the Euclidean metric values between the sets *X* and *Y*, the pair is assumed to be a ‘good’ outlier.

**Results:**

Our results showed that cluster of cities and provinces in Central Luzon (Region III), CALABARZON (Region IV-A), the National Capital Region (NCR), and Central Visayas (Region VII) are the ‘good’ outliers with respect to factors such as working population, population density, ICU beds, doctors on quarantine, number of frontliners and gross regional domestic product. Among metropolitan cities, Davao was a ‘good’ outlier with respect to demographic factors.

**Conclusions:**

Strict border control, early implementation of lockdowns, establishment of quarantine facilities, effective communication to the public, and monitoring efforts were the defining factors that helped these LGUs curtail the harm that was brought by the pandemic. If these policies are to be standardized, it would help any country’s preparedness for future health emergencies.

## Introduction

Since the emergence of the COVID-19 pandemic, the number of cases have already reached 82 million worldwide at the end of 2020. In the Philippines, the number of cases exceeded 473,000. As countries around the world face the continuing threat of the COVID-19 pandemic, national governments and health ministries formulate, implement and revise health policies and standards based on recommendations by world health organization (WHO), experiences of other countries, and on-the-ground experiences. Early health measures were primarily aimed at preventing and reducing transmission in populations at risk. These measures differ in scale and speed among countries, as some countries have more resources and are more prepared in terms of healthcare capacity and availability of stringent policies [1, 2].

During the first months of the pandemic, several countries struggled to find tolerable, if not the most effective, measures to ‘flatten’ the COVID-19 epidemic curve so that health facilities will not be overwhelmed [3, 4]. In responding to the threat of the pandemic, public health policies included epidemiological and socio-economic factors. The success or failure of these policies exposed the strengths or weaknesses of governments as well as the range of inequalities in the society [5, 6].

As national governments implemented large-scale ‘blanket’ policies to control the pandemic, local government units (LGUs) have to consider granular policies as well as real-time interventions to address differences in the local COVID-19 transmission dynamics due to heterogeneity and diversity in communities. Some policies in place, such as voluntary physical distancing, wearing of face masks and face shields, mass testing, and school closures, could be effective in one locality but not in another [7–9]. Subnational governments like LGUs are confronted with a health crisis that have economic, social and fiscal impact. While urban areas have been hot spots of the COVID-19 pandemic, there are health facilities that are already well in placed as compared to less developed and deprived rural communities [10]. The importance of local narratives in addressing subnational concerns are apparent from published experiences in the United States [11], China [12, 13], and India [14].

**Table 1:** Epidemiological, economic and demographic factors considered in the study

Factor	Definition
COVID-19 cases	Cumulative COVID-19 cases in the Philippines (as of 10 November 2020)
	from the DOH Data Drop [19]
COVID-19 deaths	Cumulative COVID-19 deaths in the Philippines (as of 10 November 2020)
	from the DOH Data Drop [19]
Population	Forecasted 2020 locality population size based
	from the 2015 Philippine Statistics Authority Census [20]
Population Density	Forecasted 2020 locality population density based
	from the 2015 Philippine Statistics Authority Census [20]
Working Population	Forecasted 2020 locality working population size (21 - 49 yrs old)
	based from the 2015 Philippine Statistics Authority Census [20]
Senior Population	Forecasted 2020 locality senior population size (70+ yrs old)
	based from the 2015 Philippine Statistics Authority Census [20]
Mobility	Percentage change in activity at each location category
	compared to that on baseline days before the advent of COVID-19
	(a 5-week period running from 3 January 2020 to 6 February 2020).
	We consider retail and recreation, grocery and pharmacy, parks,
	transit stations, workplaces and residential areas [21].
Doctors on Quarantine	Cumulative number of doctors in quarantine (as of 10 November 2020)
	at home or in a facility due to COVID-19 exposure
	(close contact, suspect, probable, confirmed) [19].
Nurses	Cumulative number of nurses in quarantine (as of 10 November 2020)
	at home or in a facility due to COVID-19 exposure
	(close contact, suspect, probable, confirmed) [19].
Frontliners	Cumulative number of other frontliners currently in quarantine
	(as of 10 November 2020) at home or in a facility due to COVID-19
	exposure (close contact, suspect, probable, confirmed) [19].
No. of ICU Beds	Total number of ICU Beds (as of 10 November 2020) [19].

In the Philippines, the Inter-Agency Task Force on Emerging Infectious Diseases (IATF) was convened by the national government in January 2020 to monitor a viral outbreak in Wuhan, China. The first case of local transmission of COVID-19 was confirmed on March 7, 2020. Following this, on March 8, the entire country was placed under a State of Public Health Emergency. By March 25, the IATF released a National Action Plan to control the spread of COVID-19. A community quarantine was initially put in place for the national capital region (NCR) starting March 13, 2020 and it was expanded to the whole island of Luzon by March 17. The initial quarantine was extended up to April 30 [5, 15]. Several quarantine protocols were then implemented based on evaluation of IATF:

- Community Quarantine (CQ) refers to restrictions in mobility between quarantined areas.
- In Enhanced Community Quarantine (ECQ), strict home quarantine is implemented and movement of residents is limited to access essential goods and services. Public transportation is suspended. Only economic activities related to essential and utility services are allowed. There is heightened presence of uniformed personnel to enforce community quarantine protocols.
- Modified Enhanced Community Quarantine (MECQ) is implemented as a transition phase between ECQ and GCQ. Strict home quarantine and suspension of public transportation are still in place. Mobility restrictions are relaxed for work-related activities. Government offices operates under a skeleton workforce. Manufacturing facilities are allowed to operate with up to 50% of the workforce. Transportation services are only allowed for essential goods and services.
- In General Community Quarantine (GCQ), individuals from less susceptible age groups and without health risks are allowed to move within quarantined zones. Public transportation can operate at reduced vehicle capacity observing physical distancing. Government offices may be at full work capacity or under alternative work arrangements. Up to 50% of the workforce in industries (except for leisure and amusement) are allowed to work.
- Modified General Community Quarantine (MGCQ) refers to the transition phase between GCQ and the New Normal. All persons are allowed outside their residences. Socio-economic activities are allowed with minimum public health standard.

LGUs are tasked to adopt, coordinate, and implement guidelines concerning COVID-19 in accordance with provincial and local quarantine protocols released by the national government [16].

In this study, we identified economic and demographic factors that are correlated with epidemiological metrics related to COVID-19, specifically to the number of infected cases and number of deaths [17, 18]. At the regional, provincial, and city levels, we investigated the localities that differ with the other localities, and determined the possible reasons why they are outliers compared to the average practices of the others.

## Methods

We categorized the data into economic, health, and demographic components (See Table 1). In the economic setting, we considered the number of people employed and the number of work hours. The number of health facilities provides an insight into the health system of a locality. Population and population density, as well as age distribution and mobility, were used as the demographic indicators. The data (as of November 10, 2020) from these seven factors were analyzed and compared to the number of deaths and cumulative cases in cities, provinces or regions in the Philippines to determine the outlier.

**Table 1:** Epidemiological, economic and societal factors considered in the study (*Continued*)

Factor	Definition
Employment	Employment Rate (as of 10 November 2020) [22].
Mean Hours Worked	The average work hours of an employee in a week
	(on 10 November 2020) [22].
GRDP	Gross Regional Domestic Product (as of 10 November 2020) [23]

The Philippine government’s administrative structure and the availability of the data affected its range for each factor. Regional data were obtained for the economic component. For the health and demographic components, data from cities and provinces were retrieved from the sources. Due to the NCR exhibiting the highest figures in all key components, an investigation was conducted to identify an outlier among its cities. The *z*-transform

$$ z=\frac{x-\mu}{\sigma} $$

where *x* is the actual data, *μ* is the mean and *σ* is the standard deviation were applied to normalize the dataset. Two sets of normalized data *X* and *Y* were compared by assigning to each pair (*x*,*y*), where *x*∈*X* and *y*∈*Y*, its Euclidean metric *d* given by *d*=(*x*−*y*)^2^. Here, the *Y*’s are the number of COVID-19 cases and deaths, and *X*’s are the other demographic indicators. Since 95% of the data fall within two standard deviations from the mean, this will be the threshold in determining an outlier. This means that if a data pair (*x*,*y*) exceeds, by two standard deviations, the mean of the Euclidean metric values between the sets *X* and *Y*, the pair is assumed to be an outlier.

To identify a good outlier, a bias computation was performed. In this procedure, *Y* represents the normalized data set for the number of deaths or the number of cases while *X* represents the normalized data set for every factor that were considered in this study. The bias is computed using the metric

$$(x-y)\times 100$$

for all *x* in *X* and *y* in *Y*. To categorize a city, province, or region as a good outlier, the bias corresponding to this locality must exceed two standard deviations from the mean of all the bias computations between the sets *X* and *Y*.

## Results and discussion

**Fig. 1:**
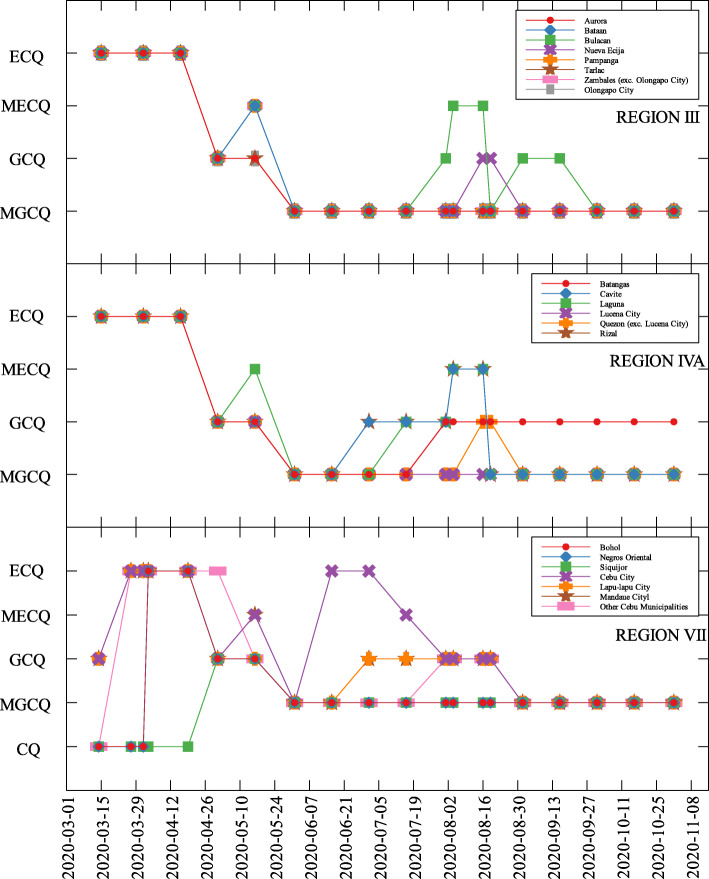
COVID-19 community quarantines in Regions III, IVA and VII

The data used were the reported COVID-19 cases and deaths in the Philippines as of November 10, 2020 which is 240 days since community lockdowns were implemented in the country. Figure 1 shows the different lockdowns implemented per province since March 15. It can be seen that ECQ was implemented in Luzon and major cities in the country in the first few weeks since March 15, and slowly eased into either GCQ or MGCQ as time progressed. By August, the most stringent lockdown was MECQ in the National Capital Region (NCR) and some nearby provinces. Places under MECQ on September were Iloilo City, Bacolod City, and Lanao del Sur, with the last province as the lone community to be placed under MECQ the month after. By November 1, 2020, communities were either placed under GCQ or MGCQ.

### Comparison of economic, health, and demographic components and COVID-19 parameters

**Fig. 2:**
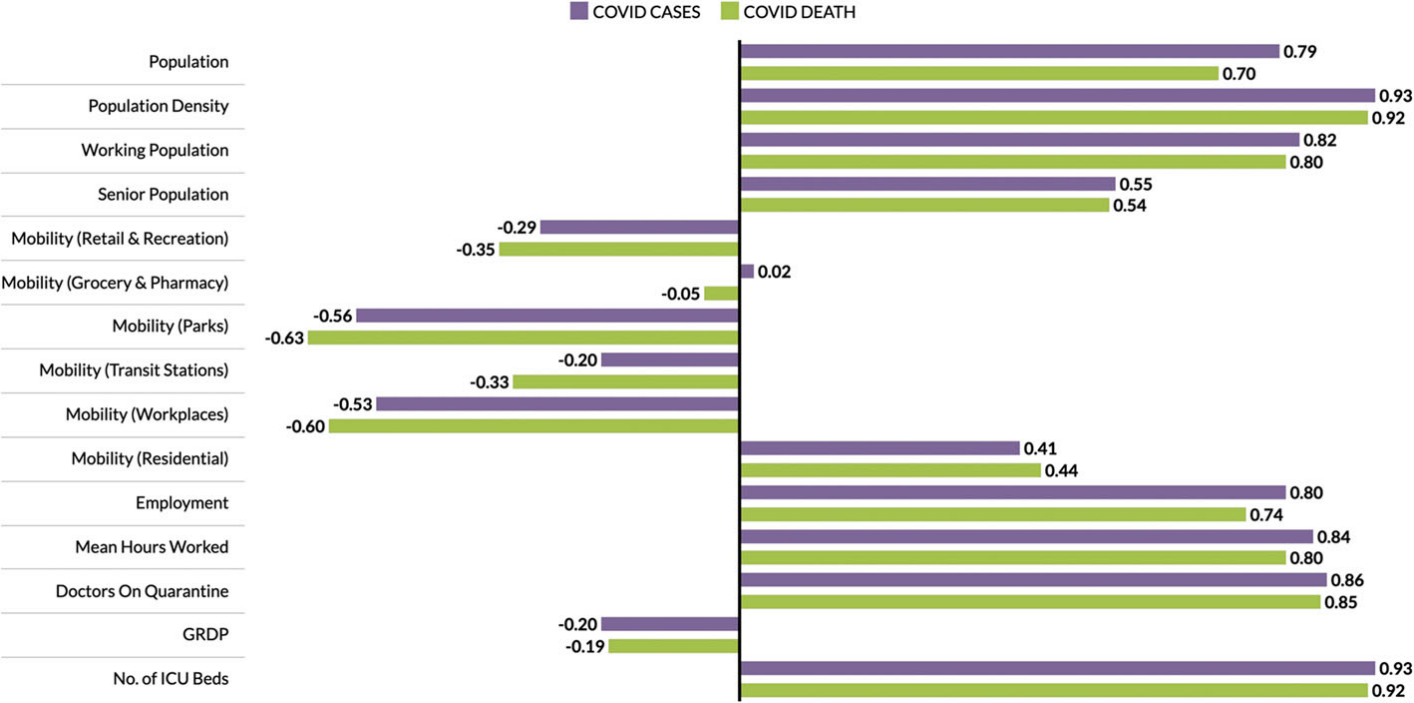
Correlation of components to COVID-19 cases and deaths at the regional level

The economic, health and demographic components were compared to COVID-19 cases and deaths. These comparisons were done for different community levels (regional, provincial, city/metropolitan) (See Tables 2, 3, and 4). Figure 2 summarizes the correlation of components to COVID-19 cases and deaths at the regional level. In all components, correlations with other parameters to both COVID-19 cases and deaths are close. Every component except Residential Mobility and GRDP have slightly higher correlation coefficient for COVID-19 cases as compared to COVID-19 deaths.

**Table 2:** Correlation table for NCR data

	Covid cases	Covid death
Population	0.970	0.945
Population Density	0.358	0.411
Working Population	0.969	0.926
Senior Population	0.970	0.945
No. of ICU Beds	0.958	0.933

**Table 3:** Correlation table for metropolitan data

	Covid cases	Covid death
Population	0.865	0.765
Population Density	0.603	0.437
Working Population	0.998	0.981
Senior Population	0.993	0.990
Mobility (Retail and Recreation)	-0.258	-0.267
Mobility (Grocery and Pharmacy)	0.137	0.112
Mobility (Parks)	-0.149	-0.136
Mobility (Transit Stations)	-0.533	-0.591
Mobility (Workplaces)	-0.499	-0.575
Mobility (Residential)	0.119	0.223

**Table 4:** Correlation table for provincial data

	Covid cases	Covid death
Population	0.851	0.697
Population Density	0.828	0.446
Working Population	0.874	0.803
Senior Population	0.737	0.728
No. of ICU Beds	0.666	0.636

Among the components, the number of ICU beds component has the highest correlation with COVID-19 parameters. This makes sense as this is one of the first-degree measures of COVID-19 transmission. Population density comes in second, followed by mean hours worked and working population, which are all related to how developed the region is economy-wise. Regions having larger population density also have a huge working population and longer working hours [24]. Thus, having a huge population density implies high chance of having contact with each other [25, 26]. Another component with high correlation to the cases and deaths is the number of doctors on quarantine, which can be looked at two ways; (i) huge infection rate in the region which is the reason the doctors got exposed or are on quarantine, and (ii) lots of doctors on quarantine which resulted to less frontliners taking care of the infected individuals. All definitions of mobility and the GDP are not strongly correlated to any of the COVID-19 measures.

### Outliers

**Fig. 3:**
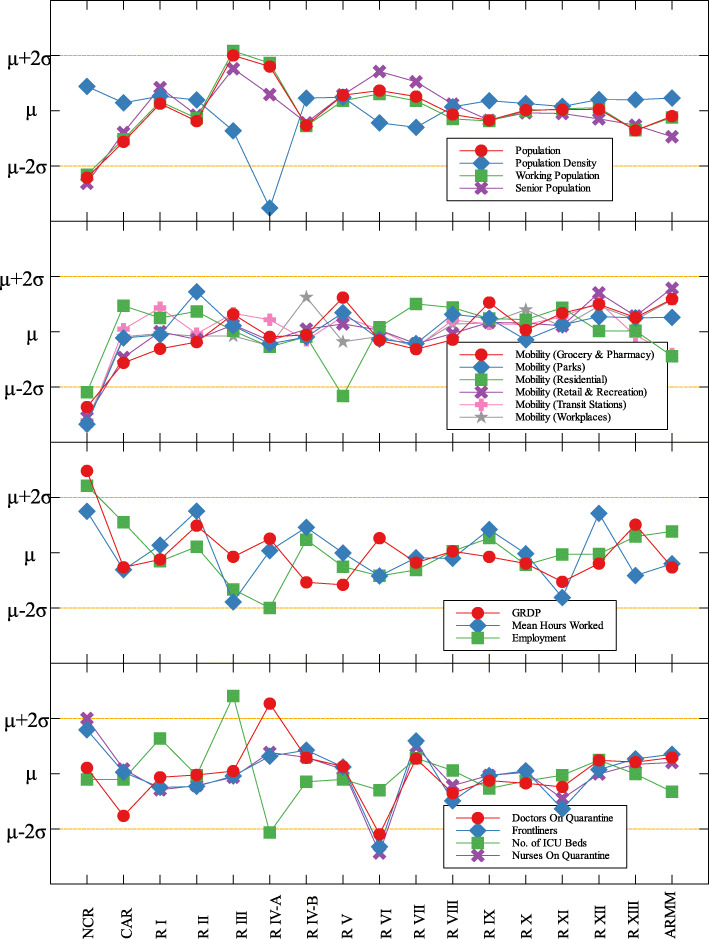
Outliers among regions in the Philippines with respect to COVID-19 cases

**Fig. 4:**
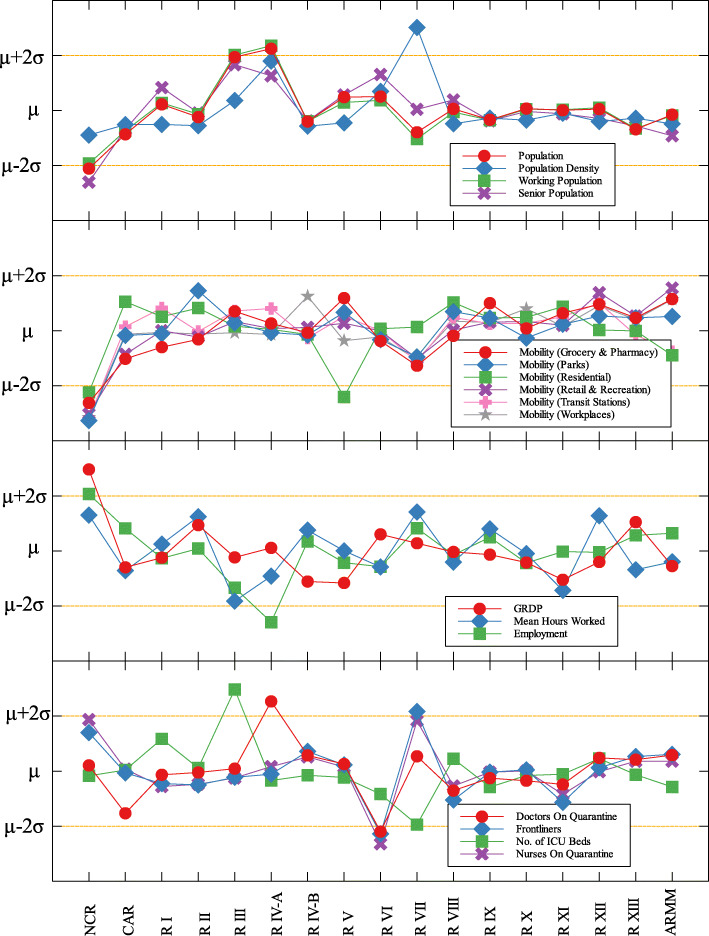
Outliers among regions in the Philippines with respect to COVID-19 deaths

In each data set, outliers were identified depending on their distance from the mean. For simplicity, we denote components that are compared with COVID-19 cases by (C) and with COVID-19 deaths by (D). The summary of outliers among regions in the Philippines is shown in Figs. 3 and 4. Data is classified according to groups of component. In each outlier region, non-pharmaceutical interventions (NPI) implemented and their timing are identified.

**Fig. 5:**
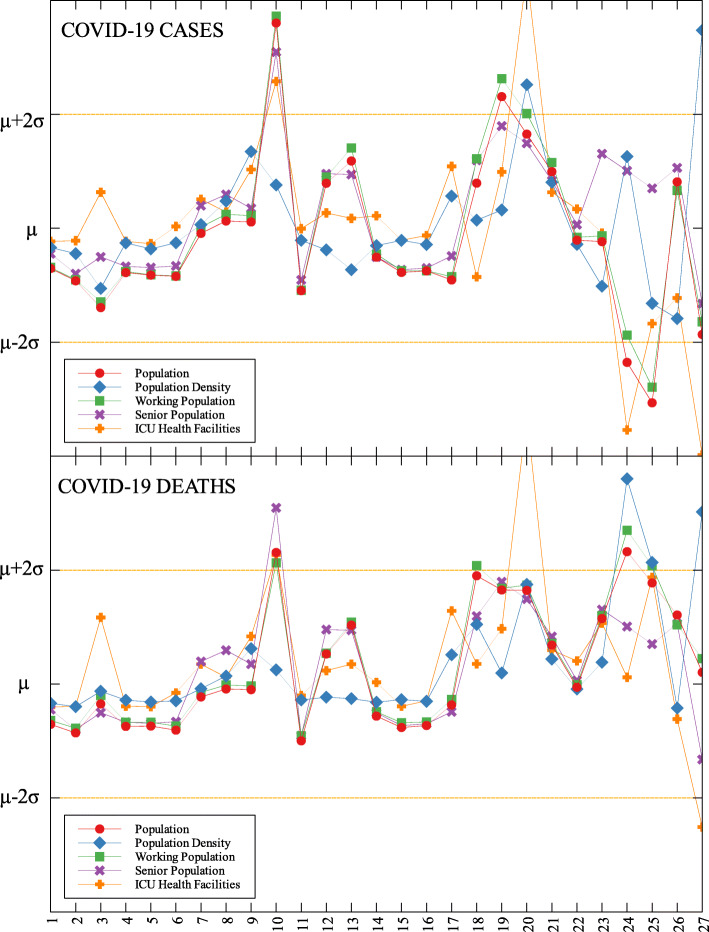
Outliers among the provinces in Luzon with respect to COVID-19 cases and deaths

Region III is an outlier in terms of working population (C) and the number of ICU beds (C) (see Fig. 5 and Table 5). This means that considering the working population of the region, the number of COVID-19 infections are better than that of other regions. Same goes with the number of ICU beds in relation to COVID-19 deaths. Region III is comprised of Aurora, Bataan, Nueva Ecija, Pampanga, Tarlac, Zambales, and Bulacan. This good performance might be attributed to their performance especially on their programs against COVID-19. As early as March 2020, the region had been under a community lockdown together with other regions in Luzon. Being the closest to NCR, Bulacan has been the most likely to have high number of COVID-19 cases in the region. But the province responded by opening infection control centers which offer free healthcare, meals, and rooms for moderate-severe COVID-19 patients [27]. They have also implemented strict monitoring of entry-exit borders, organization of provincial task force and incident command center, establishment of provincial quarantine facilities for returning overseas Filipino workers, mandated municipal quarantine facilities for asymptomatic cases, and mass testing, among others [27]. Most of which have been proven effective in reducing the number of COVID-19 cases and deaths [28].

**Table 5:** Labels for Figs. 5, 6 and 7

Label	Province	Label	Province	Label	Province
1	Abra	29	Occidental Mindoro	57	Zamboanga City
2	Apayao	30	Oriental Mindoro	58	Zamboanga Sibugay
3	Benguet	31	Palawan	59	Bukidnon
4	Ifugao	32	Romblon	60	Camiguin
5	Kalinga	33	Albay	61	Lanao del Norte
6	Mountain Province	34	Camarines Norte	62	Misamis Occidental
7	Ilocos Norte	35	Camarines Sur	63	Misamis Oriental
8	Ilocos Sur	36	Catanduanes	64	Compostela Valley
9	La Union	37	Masbate	65	Davao del Norte
10	Pangasinan	38	Sorsogon	66	Davao del Sur
11	Batanes	39	Aklan	67	Davao Occidental
12	Cagayan	40	Antique	68	Davao Oriental
13	Isabela	41	Capiz	69	Cotabato (North Cotabato)
14	Nueva Vizcaya	42	Guimaras	70	Sarangani
15	Quirino	43	Iloilo	71	South Cotabato
16	Aurora	44	Negros Occidental	72	Sultan Kudarat
17	Bataan	45	Bohol	73	Agusan del Norte
18	Bulacan	46	Cebu	74	Agusan del Sur
19	Nueva Ecija	47	Negros Oriental	75	Dinagat Islands
20	Pampanga	48	Siquijor	76	Surigao del Norte
21	Tarlac	49	Biliran	77	Surigao del Sur
22	Zambales	50	Eastern Samar	78	Basilan
23	Batangas	51	Leyte	79	Lanao del Sur
24	Cavite	52	Northern Samar	80	Maguindanao
25	Laguna	53	Samar (Western Samar)	81	Sulu
26	Quezon	54	Southern Leyte	82	Tawi-Tawi
27	Rizal	55	Zamboanga del Norte		
28	Marinduque	56	Zamboanga del Sur

**Fig. 6:**
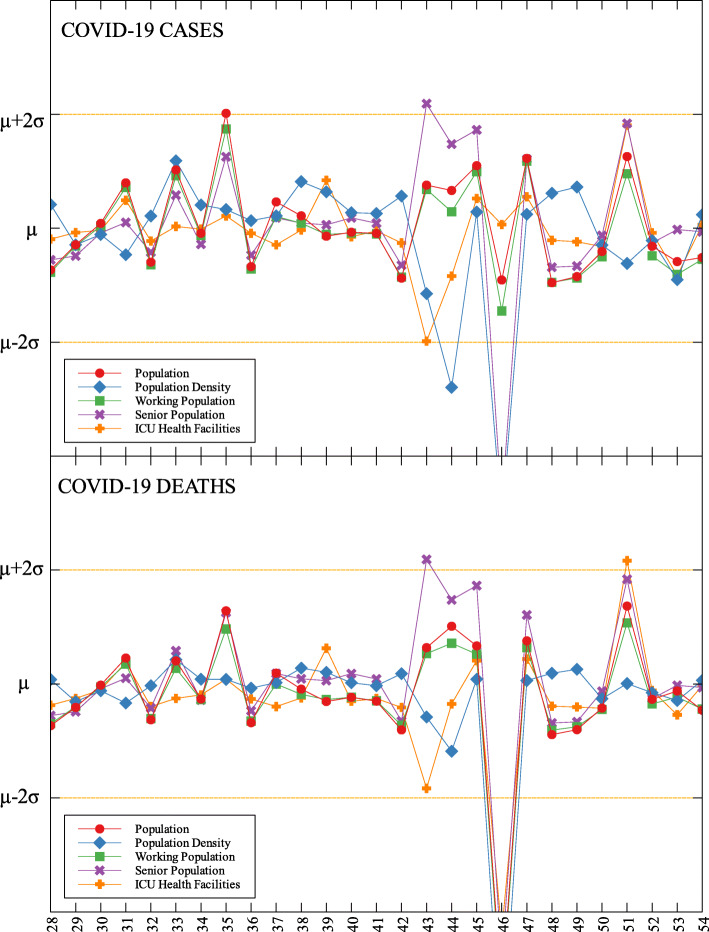
Outliers among the provinces in Visayas with respect to COVID-19 cases and deaths

**Fig. 7:**
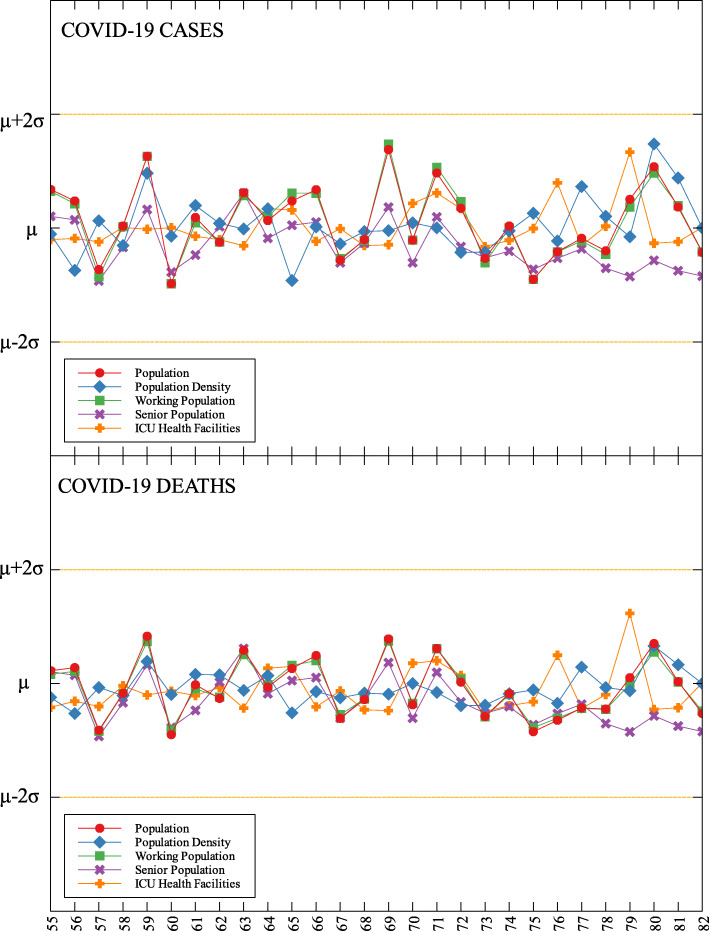
Outliers among the provinces in Mindanao with respect to COVID-19 cases and deaths

Region IV-A is an outlier in terms of population and working population (D) and doctors on quarantine (D) (see Fig. 5 and Table 5). Considering their population and working population, the COVID-19 death statistics show better results compared to other regions. Same goes with the number of doctors in the region which are in quarantine in relation to the reported COVID-19 deaths. This shows that the region is doing well in terms of decreasing the COVID-19 fatalities compared to other regions in terms of populations and doctors on quarantine. Region IV-A is comprised of Batangas, Cavite, Laguna, Quezon, and Rizal. Same with Region III, they have been under the community lockdown since March of last year. Provinces of the region such as Rizal have been proactive in responding to the epidemic as they have already suspended classes and distributed face masks even before the nationwide lockdown [29]. Despite being hit by natural calamities, the region still continue ramping up the response to the pandemic through cash assistance, first aid kits, and spreading awareness [30].

**Fig. 8:**
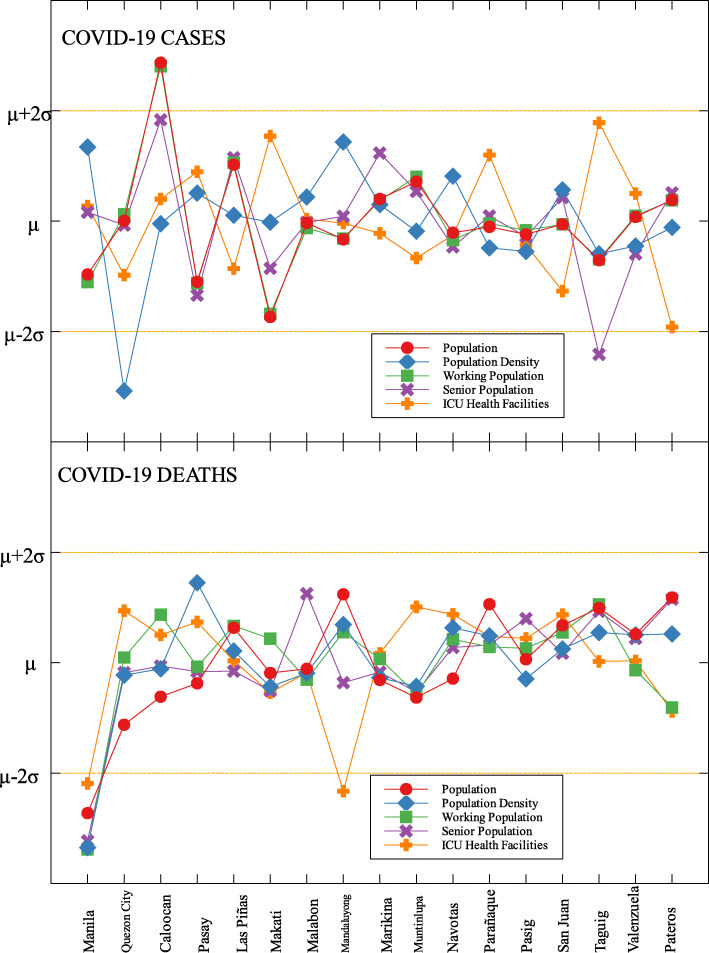
Outliers in the national capital region with respect to COVID-19 cases and deaths

An interesting result is that NCR, the center of the country and the most densely populated, is a good outlier in terms of GRDP (C) and GRDP (D). Cities in the region launched various programs in order to combat the disease. They have launched mass testings with Quezon City, Taguig City, and Caloocan City starting as early as April 2020. Pasig City started an on-the-go market called Jeepalengke. Navotas, Malabon, and Caloocan recorded the lowest attack rate of the virus. Caloocan city had good strategies for zoning, isolation and even in finding ways to be more effective and efficient. Other programs also include color-coded quarantine pass, and quarantine bands. It is also possible that NCR may just have a very high GRDP compared to other regions. A breakdown of the outliers within NCR can be seen in Fig. 8.

Region VII is also an outlier in terms of population density (D) and frontliners (D) (see Fig. 6 and Table 5). This means that given the population density and the number of frontliners in the region, their COVID-related deaths in the region is better than the rest of the country. This region consists of four provinces (Cebu, Bohol, Negros Oriental, and Siquijor) and three highly urbanized cities (Cebu City, Lapu-Lapu City, and Mandaue City), referred to as metropolitan Cebu. This significant decline may be explained by how the local government responded after they were placed in stricter community quarantine measures despite the rest of the country easing in to more lenient measures. Due to the longer and stricter quarantine in Cebu, the lockdown had a greater impact here than in other areas where restrictions were eased earlier [31]. Dumaguete was one of the destinations of the first COVID case in the Philippines [32], their local government was able to keep infections at bay early on. Siquijor was also COVID-19-free for 6 months [33]. The compounded efforts of the different provinces in the region can account for the region being identified as an outlier.

**Fig. 9:**
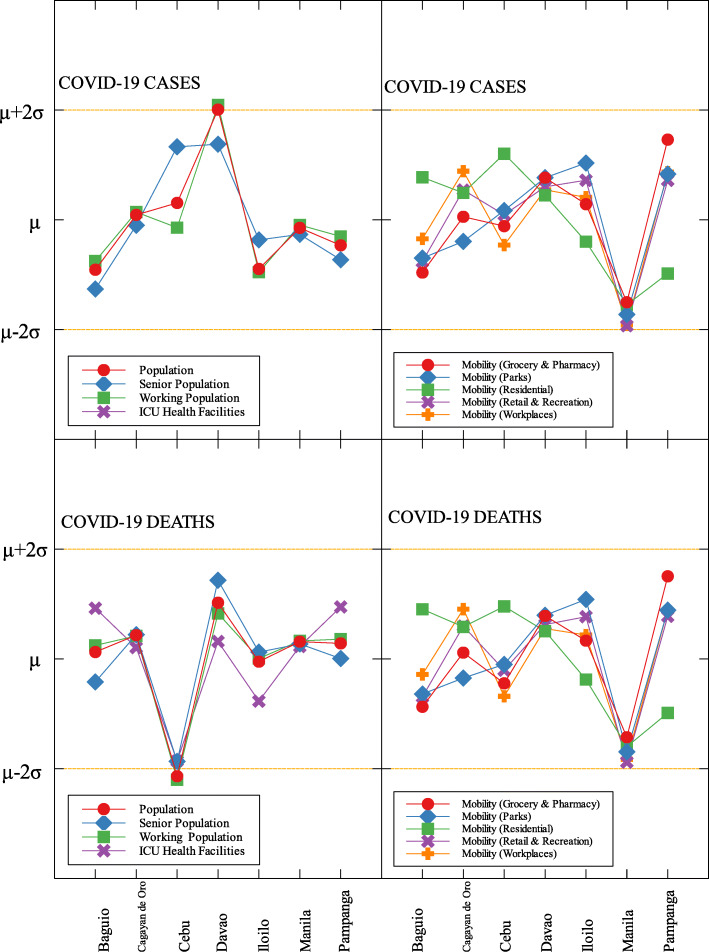
Outliers among metropolitan areas in the Philippines with respect to COVID-19 cases and deaths

Among the metropolitan cities, Davao came out as a good outlier in terms of population (C) and working population (C) (see Figs. 7, 9, and Table 5). This result may be attributed to their early campaign on consistent communication of COVID-19-related concerns to the public [34]. They were also able to set up transportation for essential workers early on [35].

## Conclusions

This study identified outliers in each data group and determined the NPIs implemented in the locality. Economic, health and demographic components were used to identify these outliers. For the regional data, three regions in Luzon and one in Visayas were identified as outliers. Apart from the minimum IATF recommended NPIs, various NPIs were implemented by different regions in containing the spread of COVID-19 in their areas. Some of these NPIs were also implemented in other localities yet these other localities did not come out as outliers. This means that one practice cannot be the sole explanation in determining an outlier. The compounding effects of practices and their timing of implementation are seen to have influenced the results. A deeper analysis of daily data for different trends in the epidemic curve is considered for future research.

## Correlation tables, outliers and community quarantine timeline

## Data Availability

The datasets used and/or analyzed during the current study are available from the corresponding author on reasonable request.
